# High-Efficiency Production of *Auricularia polytricha* Polysaccharides Through Yellow Slurry Water Fermentation and Its Structure and Antioxidant Properties

**DOI:** 10.3389/fmicb.2022.811275

**Published:** 2022-02-03

**Authors:** Zhengbin Yang, Yuedan Hu, Jiangli Wu, Jingui Liu, Furong Zhang, Hongya Ao, Yong Zhu, Laping He, Wei Zhang, Xuefeng Zeng

**Affiliations:** ^1^School of Liquor and Food Engineering, Guizhou University, Guiyang, China; ^2^Guizhou Provincial Key Laboratory of Agricultural and Animal Products Storage and Processing, Guiyang, China; ^3^Key Laboratory of Animal Genetics, Breeding and Reproduction in the Plateau Mountainous Region, Ministry of Education, Guiyang, China; ^4^College of Food Science and Engineering, Wuhan Polytechnic University, Wuhan, China

**Keywords:** yellow slurry water, polysaccharides, *Auricularia polytricha*, structure, antioxidant activity

## Abstract

Yellow slurry water is a kind of nutrient-rich wastewater of tofu. Firstly, the medium of yellow slurry was optimized. Then, APP40, APP60, and APP80 were obtained by sedimentation with different concentration of ethanol (40, 60, and 80%). The physicochemical properties and primary structures of the three polysaccharides were studied by high performance anion exchange chromatography (HPAEC), high performance gel filtration chromatography (HPGFC), scanning electron microscope (SEM), atomic force microscope (AFM), and Fourier transform infrared (FT-IR) spectrometer. Finally, the effects of three polysaccharides on antioxidation activity were studied. According to the experimental optimization the results, the biomass and the production of *Auricularia polytricha* Polysaccharides (APPS) reached the peak, and they were 13.5 ± .655 and 9.42 ± .253 g/L (*p* < .05). The SEM and the AFM showed that the height of APP80 gradually increased from 31.1 to 46.7 nm and from APP40 to APP80. The particle size of APP80 increased, the pores decrease or even disappear, and the molecules begin to aggregate. The FT-IR spectrum analysis showed that the three polysaccharides possessed key functional groups. The carbohydrate content of APP40, APP60, and APP80 was 20.2, 34.25, and 31.73%. The molecular weights of APP40, APP60, and APP80 are 9.462 × 10^4^, 8.742 × 10^4^, and 8.091 × 10^4^ Da, respectively. The three polysaccharides were composed of rhamnose, galactose, glucose, mannose, and xylose but with different molar ratio. APP80 showed strong reducing ability and scavenging activity of 2,2–diphenyl–1–picrylhydrazyl (DPPH) and hydroxyl radicals through antioxidant activities evaluated *in vitro*. This study introduces a way for the effective use of yellow slurry water.

## Introduction

Yellow slurry water is high nutrition by–product in tofu processing (Wang and Serventi, 2019). During the process, it is estimated that approximately 7–10 tons of yellow slurry water will be produced for every ton of soybean processing. Due to the high moisture content and inconvenient transportation, yellow slurry water directly discharged into the natural environment, which will lead to eutrophication of water and waste of rich resources (Wang et al., 2019). Highly nutritious wastewater makes algae and bacteria grow rapidly and consume a lot of oxygen in the water environment; thereby it results in an anaerobic condition to aquatic animals and plants and finally causes large scale of death. On the other hand, it is reported that the general solid content of yellow slurry water is as high as 1%, of which protein (.4%–.5%), total sugar (1%–2%), and a variety of vitamins, organic acids, amino acids, lipids, and other organic components are in line with the nutritional environment required by the growth of multifaceted microorganisms (Guldhe et al., 2017). Due to the enriched nutrition in this yellow slurry water, it can be utilized as a suitable substrate in fermentation industry. Some efforts have been made to utilize yellow slurry water as the medium to produce various products. For example, vitamin B12 accumulated by *Propioni-bacterium freudenreichii*, lactic acid accumulated by *Streptococcus bovis*, and isoflavone aglyconelactic by *Lactobacillus plantarum B1–6* (Yuwono and Kokugan, 2007; Xiao et al., 2015; Yu et al., 2015). In addition, yellow slurry water has been found suitable for the growth of edible fungi, such as *Tremella aurantialba* and *Hericium erinaceus* (Zhang et al., 2014; Sun et al., 2020), and can lead to higher productivities of polysaccharides.

*Auricularia polytricha* polysaccharides (APPS) have a lot of biological functions, including hypoglycemic activity (Wu et al., 2014), anti–proliferative activity (Yu et al., 2014), and anti–inflammatory activity (Hou et al., 2020). At present, the research on APPS mainly concentrates on the biological activity and macromolecular structure activity of fruiting body polysaccharide, while the research on mycelium extracellular polysaccharides is rare. Furthermore, based on the nutritional environment of yellow slurry water that meets the needs of carbon sources, nitrogen sources, and trace elements for the growth of *A. polytricha*, and even contains growth factors, it can be speculated that the biological effect of exopolysaccharide can be obtained by submerged fermentation of yellow slurry water with *A. polytricha*. Although yellow slurry water is rich in nutrition, it is also deficient. It was reported that the production of microbial EPS was affected by the composition of the culture medium, growth conditions, the age of the strain, and the amount of inoculation (Chaisuwan et al., 2020). Among them, the composition of the medium, especially the carbon source and nitrogen source, had a greater impact on the growth of microbial compounds and the production of EPS (Chaisuwan et al., 2020). Therefore, it is necessary to optimize the nutritional components of yellow slurry water medium. With the development of modern food processing technology and the deepening of environmental protection concepts, such as energy saving, efficient use of waste, and reduction of waste to the environment. Therefore, the utilization of yellow slurry water to achieve its biotransformation has become a focus of many studies, which has important social significance and economic benefits.

So far, precipitation with ethanol has been used a lot for the initial purification of aqueous extracts as a simple and rapid method (Koh et al., 2009). Importantly, the concentration of ethanol has been proved related to the molecular size, structure feature, and bioactivity of the products (Xu et al., 2014). More refined purification methods include ion–exchange chromatography, which is a widely used purification technology, using gradient salt elution to separate neutral and acidic polysaccharides. Gel filtration column chromatography is commonly adopted for separating polysaccharides with different molecular weights (Ji et al., 2020, 2021; Hou et al., 2021). Three polysaccharide fractions with different molecular weight isolated from the same species exhibited totally different antioxidant activity *in vitro* (Meng et al., 2015; Li et al., 2017).

In this study, based on the previous optimization for liquid fermentation of *A. polytricha* strain, 40, 60, and 80% alcohol precipitation was used to obtain exopolysaccharides. Besides, the effects of different ethanol concentration on the functional properties, monosaccharide composition, primary structure, and antioxidant activity of polysaccharides were investigated by high performance anion exchange chromatography (HPAEC), high performance gel filtration chromatography (HPGFC), scanning electron microscope (SEM), and atomic force microscope (AFM) in order to provide technical support for the efficient preparation and scientific utilization of extracellular polysaccharide and mycelium of *A. polytricha*, and improving the development and utilization of yellow slurry water.

## Materials and Methods

### Materials and Reagents

*Auricularia polytricha No.10* strain was purchased from Jiangsu Tianda Institute of Edible Fungi (Jiangsu, China).

Bovine serum albumin (BSA), Coomassie brilliant blue G–250, and ascorbic acid were obtained from Aladdin Reagent Co., Ltd. (Shanghai, China). Concentrated sulfuric acid, phosphoric acid, salicylic acid, trichloroacetic acid (TFA), anhydrous alcohol, acetone, anhydrous ether, glucose, sucrose, fructose, maltose and lactose, urea, peptone, ammonium sulfate, yeast and corn starch, neutral protease, 30% hydrogen peroxide (H_2_O_2_), phenol, Vitamin C (Vc) sodium hydroxide, ferrous sulfate (FeSO_4_), ferric chloride (FeCl_3_), sodium dihydrogen phosphate, disodium hydrogen phosphate, and potassium ferricyanide [K_3_Fe(CN)_6_] were purchased from Sinopharm Chemical Reagent Co., Ltd. (Shanghai, China). The monosaccharide Standards including fucose (Fuc), rhamnose (Rha), arabinose (Ara), mannose (Man), galactose (Gal), glucose (Glc), xylose (Xyl), fructose (Fru), galacturonic acid (GalA), glucuronic acid (GlcA), 2,2–diphenyl–1–picrylhydrazyl (DPPH), and potassium bromide (KBr, Spectrum pure grade) were purchased from Sigma Chemical Co. (St. Louis, MO, United States). All chemicals and solvents used in the current work were of analytical grade.

### Culture of *Auricularia polytricha* Strains

*Auricularia polytricha No.10* (AP–10) was cultured in potato glucose agar (PDA), stored at 4°C, and subcultured once a month. Besides, the slant seed culture was performed on the PDA enriched medium (PDA + KH_2_PO_4_ + MgSO_4_) at 26°C for 8 days. Then, the seed was used for liquid seed culture. After high pressure sterilization at 121°C for 30 min, the seed culture medium was cooled to room temperature, and the tilted seed culture of 1 cm^2^ was selected with the sterile inoculation ring under sterile conditions. Subsequently, two pieces of slant seed cultures were inoculated into a 250 ml flask containing 100 ml flask containing 100 ml of the initial fermentation medium containing (g/L): glucose 30, peptone 3, KH_2_PO_4_ 2, and MgSO_4_ 1 at 25°C without shaking for 24 h, and then at 160 rpm for 5 days.

### Optimization for High Density Culture of *Auricularia polytricha* (AP–10) Based on Yellow Slurry Water

#### Preparation of Yellow Slurry Water

The laboratory simulated yellow slurry water produced by the tofu factory. The process mainly involves screening, cleaning, soaking, beating, filtering, boiling, spotting, and pressing of soybeans. Then, the filtered water is called yellow slurry water, whose general element composition is 260–800 mg/L of total nitrogen, 20–50 mg/L of total phosphorus, 700–2,000 mg/L of total carbohydrates, 1,000–1,500 mg/L of total protein, 350–800 mg/L of reducing sugar, 600–700 mg/l of Na, 500–1,000 mg/L of K, 150–200 mg/L of Mg, 40–100 mg/L of Ca, and 1–3 mg/L of Fe/Zn (Wang et al., 2018, 2019; Sun et al., 2020).

#### Determination of Biomass and APPS Yields

The biomass of AP–10 was expressed by dry cell weight. The fermentation broth of the AP–10 strains was placed in the Brinell funnel and filtered until there was no dripping water. Next, they were placed at −20°C for 24 h and freeze-dried. After filtration, the supernatant was concentrated to 1/10 of the original volume, and four times the volume of anhydrous ethanol was added. The supernatant was refrigerated at 4°C for 16 h, treated at 6,000 r/min, centrifuged for 10 min, washed with acetone and ether for three times, dried at 60°C for constant weight, and then averaged by electronic analysis balance.

#### Optimization of AP–10 Strain Fermentation for Biomass and APPS Yields

As the nutrient content of yellow slurry water is not enough for the continuous growth of AP–10 strains, it is of necessity to add an appropriate number of nutrients for the growth of *A. polytricha* strains. Additionally, shake flasks of 250 ml were used to culture AP–10, while the seed medium was the PDA enriched medium, and the initial medium was yellow slurry water with 30 g/L glucose, 3 g/L peptone, 2 g/L KH_2_PO_4_, and 1 g/L MgSO_4_. Besides, the initial fermentation conditions were 10% inoculum, 25°C, and pH 5.0, with a shaking speed of 170 rpm for 3 days (Xu and Yun, 2003). To be specific, initially, the optimal fermentation period was determined. Then, the effects of temperature (15, 18, 20, 23, 26, 28, 32, and 35°C), initial pH (3–9), and the inoculum amount (1, 5, 10, 15, and 20%) on APPS yields and biomass of AP–10 were investigated, followed by the medium components being optimized. After that, five kinds of carbon sources (glucose, sucrose, fructose, maltose, and lactose) and six kinds of nitrogen sources (urea, peptone, ammonium sulfate, yeast, and corn starch) were chosen for AP–10 fermentation, with KH_2_PO_4_ (1, 1.5, 2, 2.5, 3, and 3.5 g/L) and MgSO_4_ (1, 1.5, 2, 2.5, 3, and 3.5 g/L).

### Preparation of Polysaccharides Precipitated by Different Ethanol Concentration

The extraction method refers to (Saravanakumar et al., 2021; Park et al., 2022) extraction of fungal exopolysaccharide. Firstly, the filtrate was filtered out of the liquid medium with filter paper and concentrated to one tenth of the original volume under vacuum condition. Then, the concentrated filtrate was mixed with four times volume of 95% ethanol, which was cultured at room temperature for 4°C overnight, and incubated at 6,000 r/min for 10 min. Next, the precipitate was washed with acetone and ether three times. Besides, the dried samples were prepared into 1% (m/v) polysaccharide solution, and 3% polysaccharide neutral protease was added, while pH was adjusted to 7 and reacted at 42°C for 3 h. After being reacted, the samples were cooled to room temperature and shaken for 10 min with Sevag solution reagent for 6,000 r/min for 10 min in order to collect the upper aqueous phase. Here, it should be mentioned that the above steps were repeated until there was no obvious white viscous material between the aqueous phase and the organic phase, followed by being concentrated, dialyzed (8,000–12,000) kDa. Finally, the obtained samples (polysaccharides) were precipitated at the ethanol concentration of 40, 60, and 80%, which were named APP40, APP60, and APP80, respectively.

### Characterization and Composition of the Polysaccharide Constituent Monosaccharides and Molecular Weights

#### Chemical Properties

The total carbohydrate content was determined by employing the phenol–sulfuric acid method (DuBois et al., 2002), and protein was measured with the Bradford method (Yuan et al., 2019) with BSA as a standard, while ζ – potential was confirmed by the Laser Particle Size Analyzer (Malvern Instruments, Worcestershire, United Kingdom). Besides, distilled water was used as the dispersant.

#### Monosaccharide Composition Analysis

Around 5 mg of polysaccharide samples were weighed in a 20 ml calibration tube with the plug. Meanwhile, 1 ml of 2 mol/L TFA wad added and hydrolyzed in an oven at 121°C for 2 h. Then, the volume was adjusted to 50 ml with water and filtered with .45 μm microporous membranes for injection analysis. At the same time, the monosaccharide composition was determined by HPAEC system equipped with a pulsed amperometric detector (PAD), a Dionex ICS–5000 equipped with a CarboPacTM PA20 column (3 mm × 150 mm), in line with the method of (Xie et al., 2013). Test conditions are shown in Table 1.

**Table 1:** HPLC mobile phase conditions.

	Water (%)	250 mM NaOH (%)	1 M NaAc (%)
0 min	98	2.0	0
21 min	98	2.0	0
21.1 min	93	2.0	5.0
30 min	78	2.0	20
30.1 min	20	80	0
50 min	20	80	0

#### Molecular Weight Analysis

The MWs of purified polysaccharide fractions were determined by HPGFC using a Waters HPLC system (1,525 pump and 2,414 refractive index detector, United States) equipped with a size exclusion Ultrahydrogel™ Linear 300 mm × 7.8mmid × 2 column with .1 mol/L Sodium nitrate as the mobile phase at a flow rate of .9 mL/min and the column temperature of 45°C. As for sample preparation, the sample is dissolved in the mobile phase and filtered by the microporous filter membrane, while the standard employed in the molecular weight calibration curve is (MW135350, MW36800, MW9750, MW2700, and MW180).

### Atomic Force Microscopy

The AFM (Dimension Icon, Bruker, United States) bases on the repulsive force between the probe and the sample surface to obtain the atomic level image of the sample surface and the aggregation degree of the sample in water. It should be pointed out that the sample was firstly immersed in distilled water (1 μg/ml) for 30 min. Then, after ultrasonic treatment and being dried at 105°C for .5 min, it was analyzed.

### Fourier Transform Infrared Spectroscopy Analysis

The FT-IR spectra of the freeze–dried APP40, APP60, and APP80 were determined with a PerkinElmer FTIR spectrometer (Frontier, PerkinElmer, Boston, MA, United States). All spectra were obtained using PerkinElmer Spectrum 10.4.2.279 software (Frontier, PerkinElmer, Boston, MA, United States) in the frequency ranging between 4,000 and 500 cm^−1^ (wave number) by adopting the attenuated total reflectance technique. Furthermore, it should be mentioned that each sample was measured in triplicate.

### X–Ray Diffraction Analysis

The dialyzed samples were taken at −20°C and then freeze-dried by a freeze drier (VaCo5–ll–D, Zirbus technology GmbH Hilfe Gottes, Germany). Subsequently, the crystalline structure of the three samples was evaluated using a powder X–ray diffractometer (Empyrean, PANalytical B.V., Netherlands) at the operating voltage and current of 45 kV and 40 mA, respectively, when the diffraction intensities were swept on the powder from 10 to 55° (2θ angle range) with a step size of .013° and a step rate of .3 s per step.

### Scanning Electron Microscopy

The pulverized freeze–dried APP40, APP60, and APP80 samples were spread on a SEM stub with sticky double–side carbon tape and coated with a thin gold layer using a coater. Then, examination was performed by the SEM (SU8010, HITACHI, Japan) operated at 10.0 kV high voltage.

### Antioxidant Activity Assays

#### Scavenging Assays of the DPPH Radical

2,2–diphenyl–1–picrylhydrazyl is a stable radical in organic solvents, which has an electron and can accept an electron or a hydrogen ion. In the presence of the free radical scavenger, the single electron of DPPH is captured, making the color of DPPH become lighter and the absorbance value at the maximum light absorption wavelength decrease linearly.

2,2–diphenyl–1–picrylhydrazyl radical scavenging activity was determined using the slightly modified method of (Rozi et al., 2020). To be specific, the freshly prepared DPPH–methanol solution (.2 mmol/L) was added with 100 μl different concentration of APP40, APP60, and APP80. Next, the absorbance at 517 nm was measured with a microplate reader, while *V*_c_ was used as a reference material. All tests were performed in triplicate. Besides, the scavenging activity was calculated as follows:


$$\mathrm{Scavenging activity}\;\left(\%\right)=\left[\left(A_{1}-A_{2}\right)/A_{0}\right]\times 100$$

where *A*_1_, *A*_2_, and *A*_0_ represent the absorbance of samples with DPPH–methanol solution, samples with methanol, and distilled water with DPPH–methanol solution, respectively.

#### Hydroxyl Radical Scavenging Assays

Hydroxyl radical–scavenging activity was measured in accordance with Smirnoff’s work (Zhang et al., 2012). Therefore, .5 ml of FeSO_4_ (1.5 mm) was mixed with .35 ml of H_2_O_2_ (6 mM), .15 ml of sodium salicylate (20 mM), and 1.0 ml of the sample (.1–4.0 mg/ml). Then, the mixture was incubated at 37°C for 1 h. In addition, the absorbance of hydroxysalicylic acid complex was measured at 562 nm, while *V*_c_ was used as positive control. The formula of antioxidant activity is expressed as follows:


$$\mathrm{Scavenging effect}\;\left(\%\right)=\left[\left(A_{0}-A_{1}\right)/A_{0}\right]\times 100$$

where *A*_0_ refers to the absorbance of the solvent control and *A*_1_ indicates the absorbance of the test (sample or *V*_c_).

#### Reducing Power

The reducing power of APP40, APP60, and APP80 was determined by employing the previous method (Rozi et al., 2019). In brief, the different concentration of test samples (.1–4.0 mg/ml) was mixed with phosphate buffers (.1 ml, .2 mol/L, and pH 6.6) and potassium ferricyanide [K_3_Fe(CN)_6_; .1 ml, 1%], followed by the mixture being incubated at 50°C for 20 min. Then, .1 ml TFA (10%) was added for stopping the reaction, while distilled water (.4 ml) and FeCl_3_ (.1 ml, .1%) were added and stored at room temperature for 10 min. Subsequently, the absorbance was measured at 700 nm. Besides, *V*_c_ was used as the positive control.

### Statistical Analysis

All the experiments were repeated three times. The results were expressed as mean ± SD. Furthermore, Origin 2018 was used to draw a map, and SPSS 22.0 was adopted for performing statistical analysis, while ANOVA was applied to determine significant differences between data. s

## Results and Discussion

### Culture of *Auricularia polytricha*

*Auricularia polytricha-10* was placed in the fermentation bottle for fermentation for 3 days. The shape of the mycelium pellets in the control medium and yellow slurry medium was shown in Supplementary Figure 1S. The mycelium pellets were relatively large, uneven distribution and low density, and the fermentation liquid was clear in the control medium. However, the mycelium pellets were small and evenly distributed, with high density and spherical distribution in the conical flask in the medium of yellow slurry water. This further confirmed that yellow slurry water can promote culture.

### Effects of Fermentation Conditions on APPS and Biomass Production With AP–10

**Figure 1:**
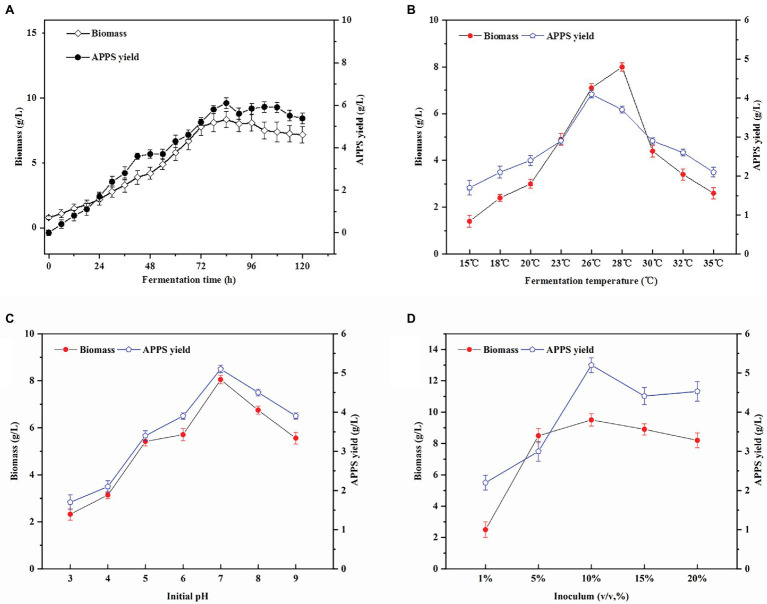
Effects of culture conditions on *Auricularia polytricha* Polysaccharides (APPS) and biomass production. **(A)** The effect of fermentation time on the production of APPS; **(B)** optimization of fermentation temperature (pH 5 and initial inoculum of 10%); **(C)** optimization of fermentation temperature (pH 5 and initial inoculum of 10%); and **(D)** optimization of fermentation initial inoculum (pH 7 and temperature of 26°C).

The different fermentation parameters, including time, temperature, and inoculum, were investigated as shown in Figure 1. In Figure 1A, within 96 h after the beginning of fermentation, the biomass and APPS yields increased linearly with fermentation time. Then, the curve entered a brief plateau, probably due to depletion of carbon sources. The yield of APPS was the highest (6.1 ± .247 g/L), which was significantly higher than that of 24 h (1.7 ± .21 g/L) and 48 h (3.7 ± .189 g/L; *p* < .05). As a result, 96 h is the best end time of fermentation.

Temperature is one of the main conditions affecting the growth of microorganisms, and each strain has its own optimal growth temperature. As presented in Figure 1B, from 15 to 26°C, the biomass of AP–10 increases significantly with temperature from (1.4 ± .27 g/L) to (7.1 ± .175 g/L; *p* < .05), reaching its maximum at 28°C. APPS production increases dramatically when the temperature increases from 23 to 26°C, and decreases dramatically when the temperature exceeds 26°C. The highest yield of mycelia does not necessarily indicate the highest level of synthetic products (polysaccharide content). Actually, this inconsistency between the synthetic products and the optimal conditions at which cells grow is not uncommon in industrial fermentation. Besides, *Aureobasidium* was applied to produce amylopectin, when the optimum growth temperature of *Aureobasidium* was 26°C and that of amylopectin synthesis was 22°C. Considering mycelial biomass and polysaccharide yields, the optimum fermentation temperature was 26°C. At this time, AP–10 biomass was 7.1 ± .175 g/L and the polysaccharide yield was 4.1 ± .095 g/L (Wu et al., 2012).

pH value is another important factor influencing cell growth and product synthesis. According to our results (Figure 1C), the AP–10 biomass grows normally at pH 4 and 8. When the initial pH was 7, the highest yield of AP–10 biomass was 5.1 ± .086 g/L, which was significantly higher than that of pH 3 (1.7 ± .19 g/L) and pH 9 (3.9 ± .079 g/L; *p* < .05). The initial pH value of the medium may affect cell structure, cell membrane properties, and nutrient absorption, thereby affecting the secretion of extracellular polysaccharides (Castillo et al., 2015). Moreover, from some previous studies, it was found that neutral pH was more suitable for basidiomycetes growth and synthesis of polysaccharides, which was consistent with our optimization results (Kim et al., 2005).

The amount of inoculation also exerted a significant effect on the fermentation process. As shown in Figure 1D, when the temperature was 26°C, the biomass increased with the increase of inoculation amount, and reached the peak when the inoculation amount was 10%. The polysaccharide yield and biomass of AP–10 did not increase significantly or even decreased when the inoculum was over 10%. The reason is that when the initial inoculum was too small, the mycelial growth would be in a long lag period, the culture time would be prolonged, the productivity would be reduced, and the mycelium could not be obtained. Differently, high initial inoculation amount not only leads to insufficient dissolved oxygen, but also causes excessive migration of metabolic waste, resulting in the reduction of metabolites (Xiong et al., 2020). Therefore, 10% inoculation amount was selected as the best inoculation amount in this study.

### Effects of the Medium Component on APPS Production With AP–10

After obtaining the optimal temperature and inoculation parameters, adjusting the composition of the medium is the main way to increase the yield of the target product.

**Figure 2:**
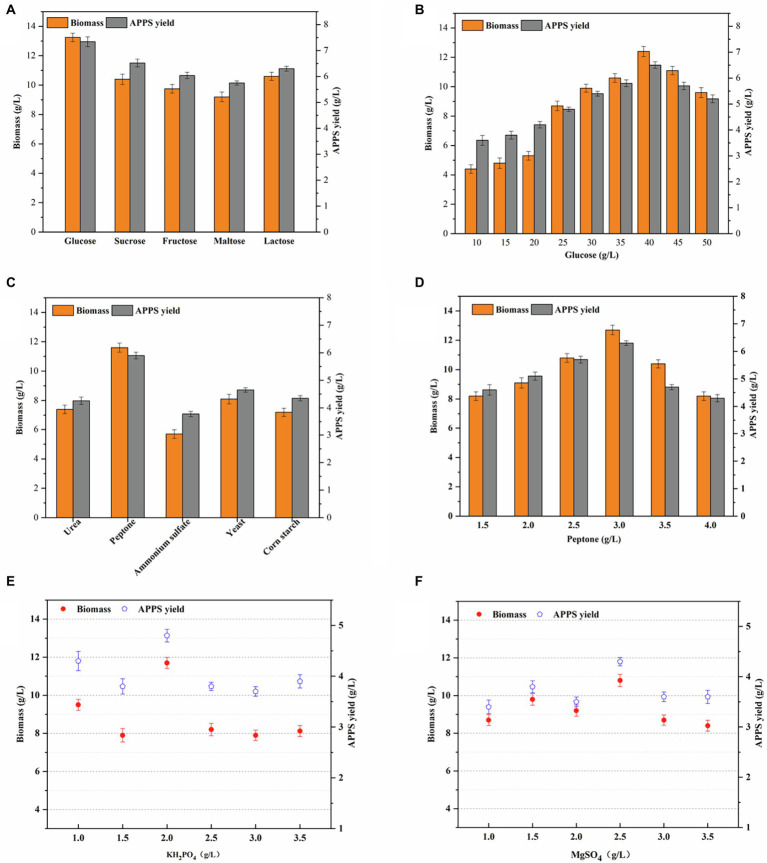
Effects of different carbon sources and nitrogen sources and inorganic saltson APPS and biomass production by AP–10 fermentation and time distribution of yellow slurry water fermentation system. **(A)**. Optimization of fermentation carbon sources (pH 7, the nitrogen source was 3 g/L peptone, the inorganic saltson was a mixture of 2.5 g/L KH_2_PO_4_, and 1 g/L MgSO_4_ for 3 days); **(B)** optimization of fermentation glucose concentration; **(C)** optimization of fermentation nitrogen sources (the carbon source was 40 g/L glucose); **(D)** optimization of fermentation peptone concentration; and **(E,F)** optimization of fermentation for the amount of inorganic saltson added.

The carbon source exerts a great influence on microbial biomass and extracellular polysaccharide production, because it not only provides nutrients for cell growth, but also offers the carbon framework for polysaccharide synthesis. Therefore, the carbon source is usually added to the screening medium in order to screen polysaccharide secreting strains (Duan et al., 2018). As shown in Figure 2A, glucose was replaced by sucrose, fructose, maltose, and galactose. The results demonstrated that glucose was still the most favorable carbon source for the growth and secretion of AP–10. Meanwhile, with the increase of glucose concentration in the medium, the biomass of AP–10, and the yield of APPS increased to a great extent. To be specific, when the glucose concentration exceeded 40 g/L, the yield of biomass and polysaccharide did not continue to increase, while as the glucose concentration was over 45 g/L, the yield of biomass and polysaccharide began to stop (Figure 2B). This can be caused by going beyond the optimal range of the carbon-nitrogen ratio. Specifically, when the C/N ratio is too large or too small, the growth of edible fungi mycelia is poor or even undeveloped. Therefore, the best carbon source is glucose, with the maximum concentration of 40 g/L and its applied yield of 6.5 ± .172 g/L (*p* < .05). Based on numerous studies, it has been found that sucrose is the main carbon source of microbial EPS synthesis (Ravella et al., 2010). Compared with (Rutering et al., 2016) with 40 g/L glucose as the best carbon source, the yield of 4.54 g/L *bacteroides* polysaccharide was better. It was reported that 2% glucose was the optimal carbon source for the fermentation of *Coprinus comatus* polysaccharide (Cao et al., 2019). It should be noted that different strains had different optimal carbon sources in different fermentation systems. Because the culture environment is yellow slurry water, the carbon source is different from that required by other strains (Nouha et al., 2018).

The nitrogen source is the source of nitrogen during the growth and differentiation of edible fungi, which provides raw materials for the synthesis of protein and nucleic acid. After determining the type and concentration of carbon sources, urea, peptone, ammonium sulfate, yeast powder, and corn powder were selected as nitrogen sources, while peptone was the best. Its mycelial biomass was 11.6 ± .312 g/L (*p* < .05), and the yield of APPS was 5.9 ± .12 g/L (*p* < .05; Figure 2D). Because *A. polytricha* is a parasitic edible fungus, it is more suitable for organic nitrogen.

KH_2_PO_4_ and MgSO_4_ are often added to the formula of many edible fungus mother species to supplement mineral nutrients. As essential additives for liquid culture of edible fungus, these two elements provide essential elements for the growth of agaric fungus and play an extremely significant role in the fermentation process. Phosphorus is the component of mycelium and plays an important role in material metabolism, whereas KH_2_PO_4_ can not only inhibit the aging of *A. polytricha*, but also does not influence its growth rate. Magnesium is the activator of many enzymes and can promote carbohydrate metabolism, nucleic acid synthesis, phosphate conversion, etc. Therefore, it is necessary to optimize these two factors. Based on the determination of carbon and nitrogen sources, KH_2_PO_4_ and MgSO_4_ were optimized, and the optimal values were 2.0 and 2.5 g/L (*p* < .05), while the mycelial biomass and APPS were 11.7 ± .294, 10.8 ± .325, 4.8 ± .125 g/L (*p* < .05), and 4.3 ± .082 g/L (*p* < .05; Figure 2C). It indicates that the high concentration of trace elements is not conducive to the synthesis of polysaccharides and may be harmful to cells (Dlugaszek, 2019). In general, metal elements have a great influence on the growth of microorganisms and the production of metabolites. Specifically, the main function of Na and Ca is to maintain the osmotic pressure inside and outside the cell and increase the enzyme activity. Zn is the functional component of the enzyme required to synthesize DNA and RNA. Fe is an enzyme activator and plays an important role in electron transfer during cell respiration. Additionally, both Cu and Zn are essential substances of fungal enzyme activity, which can enhance the growth of camphor, resulting in the induction, production, and molecular weight changes of sulfated polysaccharides (Lin et al., 2018). Furthermore, the above microelements were found in the yellow slurry water fermentation system, promoting the growth of mycelia and the secretion of metabolites (Figures 2E,F).

### Time Distribution of the Yellow Slurry Water Fermentation System

**Figure 3:**
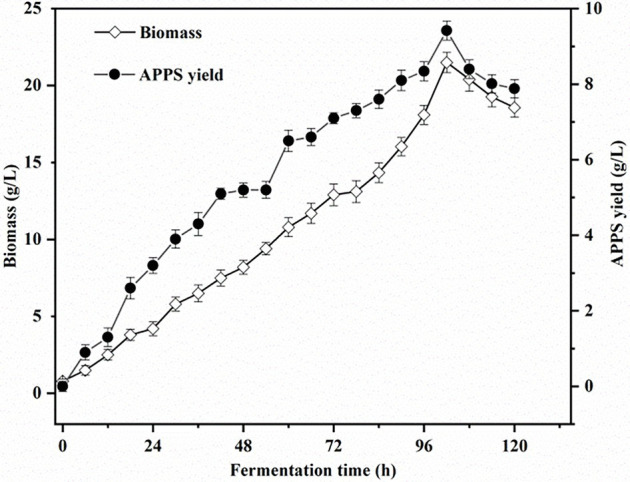
Time distribution of yellow slurry water fermentation system.

The above optimal factors (Glucose 40 g/L, Peptone 3 g/L, KH_2_PO_4_ 2.0 g/L, MgSO_4_ 2.5 g/L, fermentation temperature, initial pH, and inoculation amount were 26°C, 7, and 10%, respectively) were applied as fermentation conditions. As shown in Figure 3, the biomass and APPS production increased linearly with the fermentation time within 96 h after the start of fermentation. Then, the curve entered a transition phase peak, which may be due to the depletion of carbon sources. At this time, full use of the nutrients contained in the yellow slurry water can continue to provide energy. After that, at 102 h, the biomass and the production of APPS reached the peak, and they were 13.5 ± .655 and 9.42 ± .253 g/L (*p* < .05) accordingly. Moreover, (Xu and Yun, 2003) investigated the fermentation conditions of spore polysaccharides with *A. polytricha*. The best fermentation time was 8 days. Differently, the yellow slurry water fermentation time of *T. aurantialba* was shorter than that of ordinary fermentation, and the yield of polysaccharide was higher (Sun et al., 2020), which was consistent with the conclusion in this study. In addition, it can be speculated that there are additional nutrients and stimulating factors in the yellow slurry water culture environment to promote mycelia growth and secretion, aiming to further shorten the fermentation time and improve the yield. Then, a good example is that the number of microalgae hyphae cultured in yellow slurry water is larger than that of the mBG–11 medium (Wang et al., 2019). For the fruiting body cultivation of *Auricularia* preliminary, the general cultivation cycle is 50–70 days. Although the fermentation cycle in this study is longer than that of general bacterial fermentation, it has saved much time and cost compared with fruiting body cultivation of *A. polytricha*. It is also about developing a new way to get APPS faster.

### Characterization and Composition of the Polysaccharides

**Figure 4:**
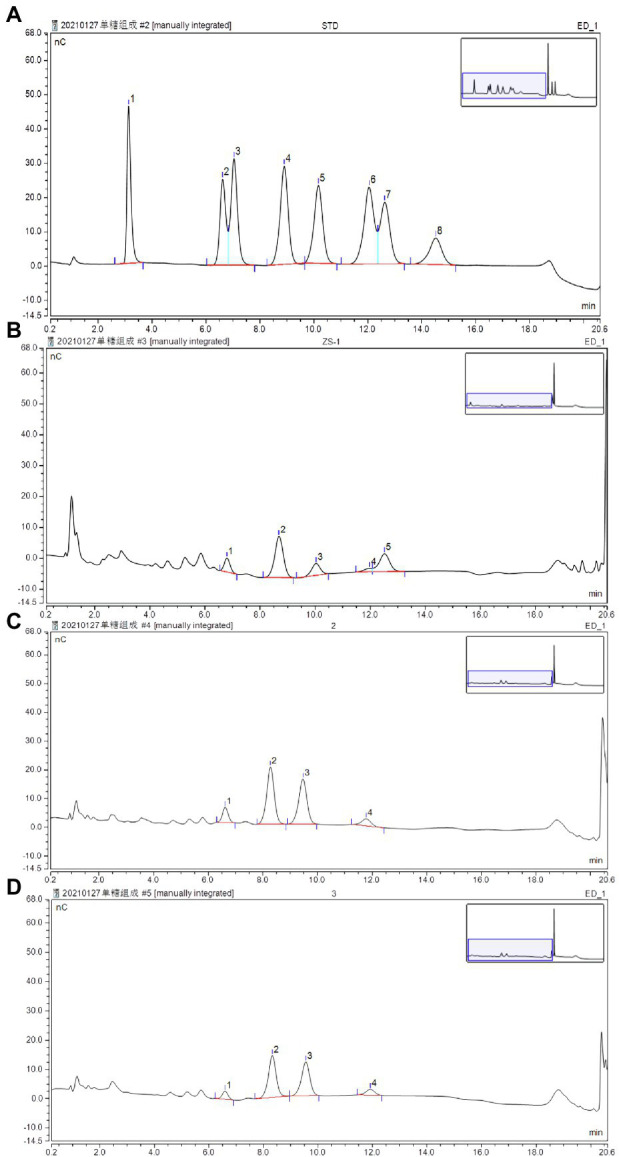
Determination of monosaccharides by HPLC using the pulsed amperometric detector (PAD) detector: **(A)** standard chromatogram of monosaccharide mix contains fucose (1), arabinose (2), rhamnose (3), galactose (4), glucose (5), xylose(6), mannose (7), and fructose (8); **(B)** determination of monosaccharides in APP40; **(C)** determination of monosaccharides in APP60; and **(D)** Determination of monosaccharides in APP80.

Monosaccharides are natural basic units, which may determine the unique structure characters and bioactivities of polysaccharides (Yuan et al., 2019). Their composition was shown in Table 2. The content of monosaccharides was different among the three polysaccharides. However, all the three kinds of polysaccharides were composed of different content ratio of rhamnose, galactose, glucose, and mannose (Figure 4).

**Table 2:** Characterization and composition of the polysaccharides.

Item	APP40	APP60	APP80
Carbohydrate (%)	20.2 ± .23^c^	34.25 ± .15^a^	31.73 ± .25^b^
Protein (%)	4.49 ± .33^a^	2.18 ± .22^b^	2.37 ± .47^b^
Yield (%)	1.43^c^	2.25^b^	5.74^a^
Mw × 10^4^(Da)	9.462	8.742	8.091
Mn × 10^4^(Da)	6.687	6.0798	5.367
Mw/Mn	1.41	1.43	1.51
ζ–potential(mV)	−13.06^a^	9.63^b^	4.23^c^
**Monosaccharide composition content ratio (%)**			
Rhamnose	10.75	8.94	6.65
Galactose	47.24	45.19	46.14
Glucose	13.34	38.89	39.84
Xylose	3.53	—	—
Mannose	25.14	6.98	7.37

Values represent mean ± SD, and superscripts a–c differ significantly (*p* < .05) among APPS.

**Figure 5:**
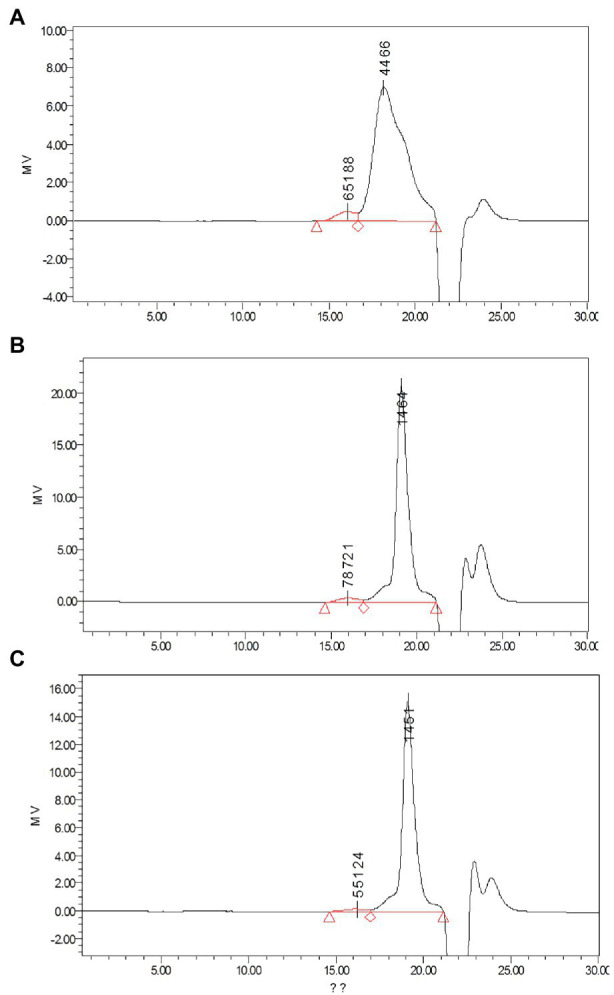
Molecular weight of the three polysaccharides. **(A)** APP40; **(B)** APP60; and **(C)** APP80.

The total yield of APPS was approximately 9.42% (w/v) of the wet material, namely, APP40 (1.43%), APP60 (2.25%), and APP80 (5.74%), which provided technical support for the further separation of this study based on (Saravanakumar et al., 2021) extracellular extraction technology of fungi. Its extraction technology is different in the use of different ethanol concentration separation of different molecular weight polysaccharides. According to the results, the carbohydrate content of APP60 was the highest (34.25%), which was significantly higher than that of APP40 (20.2%). However, the protein content of APP40 (4.49%) was obviously higher than that of APP60 (2.18%) and APP80 (2.37%). The obtained results are consistent with those of an earlier work that reports the presence of protein in the polysaccharides isolated from fungi (Park et al., 2022). In addition, the distribution of mannose in 40% ethanol concentration was higher than that in 60 and 80% ethanol concentration, while the proportion of monosaccharide in each part of polysaccharide was also different. Furthermore, with the increase of the ethanol volume fraction, as shown in Figure 5 and Table 2, the molecular weight increased from 9.462 × 10^4^ to 8.091 × 10^4^ Da. Differently, the proportion of rhamnose, a common component of polysaccharides from *A. polytricha*, decreased significantly, while that of glucose increased. Besides, only the presence of xylose in APP40 (3.53%) was similar to the effect of different extraction processes on the monosaccharide composition of polysaccharides (Yan et al., 2015; Wang et al., 2016). The change of the ethanol volume fraction exerts more influence on the molecular weight of polysaccharides in the precipitation, because the low volume fraction of ethanol precipitates is mainly high molecular weight polysaccharides, while the high volume fraction of ethanol precipitates is small molecular weight polysaccharides and oligosaccharides. The smaller the molecular weight, the freer amino groups in the molecule, which may lead to its activity enhancement. Besides, the natural polysaccharides have higher antiobesity and antidiabetic effects *in vitro*, which may be associated with their high molecular weight (Fu et al., 2019; Yuan et al., 2019), which also provides an effective idea for reducing molecular weight through ethanol concentration precipitation under better functional conditions.

Zeta potential can be used to determine the stability of polysaccharide solution. In general, the higher the Zeta potential of the dispersion system is, the greater the electrostatic repulsion between molecules is, which can make molecules more dispersed and stable between solutions. On the contrary, the lower the Zeta potential is, the smaller the repulsive force between molecules is. Besides, the aggregation between molecules is easy to occur (Tang et al., 2020). Moreover, it can be found from Table 2 that APP is anion polysaccharide, and the potentials of APP40, APP60, and APP80 are −13.06, −9.63, and −4.23 mV, respectively, indicating the stability of solution APP80 > APP60 > APP40.

#### The FT–IR Analysis

**Figure 6:**
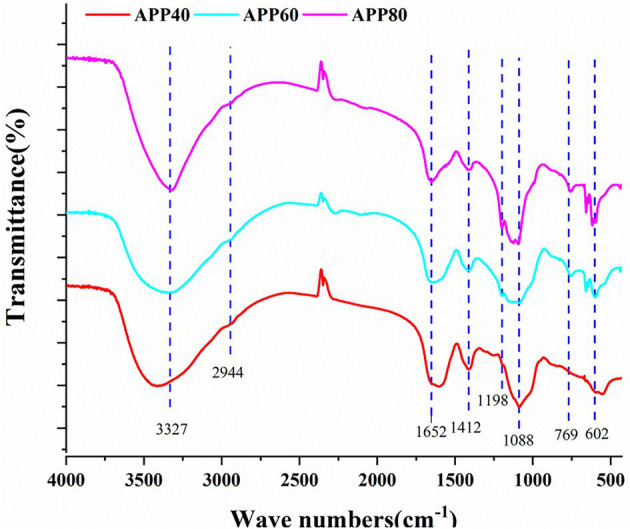
The Fourier transform infrared (FT-IR) analysis of precipitated at the ethanol concentration of 40% (APP40), precipitated at the ethanol concentration of 60% (APP60), and precipitated at the ethanol concentration of 80% (APP80).

The structure characteristics of crude polysaccharides precipitated by different volume fractions of ethanol were explored by FT-IR spectroscopy. As shown in Figure 6, the infrared spectra of APP40, APP60, and APP80 are similar. It can be found that in the wave number range of 3,700–3,100 cm^−1^, there is a peak near 3,327 cm^−1^, which is a typical O–H stretching vibration. As this peak is larger and wider, it is a typical characteristic peak of polysaccharides. Additionally, within the range of 3,000–2,800 cm^−1^, there is a weak C–H absorption peak in the region, and the peak appearing at 2,944 cm^−1^ is not very significant and caused by the asymmetric angular vibration of the C–H bond in the molecule (Guo et al., 2020), when the stretching vibration of CH, CH_2_, and CH_3_ is included (Wang et al., 2012). A group of absorption peaks in the range of 1,400–1,200 cm^−1^ were C–H variable angle vibration peaks. The above three characteristic peaks indicated that the substance was a polysaccharide. Furthermore, the strong peak at 1,652 cm^−1^ may be the asymmetric stretching vibration of the C=O group, and the vibration at 1,412 cm^−1^ may be attributed to the bending vibration of C–O or O–H, or the CH_2_ group adjacent to the C=O group. Different from that above-mentioned, the vibration in the range of 1,000–1,200 cm^−1^ (1,088 and 1,198 cm^−1^) was caused by that of the ester sugar group (C–O–C) and (C–O–H) of pyranose rings (Wang et al., 2015; Wu et al., 2015), proving that Sichuan APP40, APP60, and APP80 all contained pyranose.

#### XRD Analysis

X–ray diffraction (XRD) is an analytical method of reflecting the crystallization characteristics and crystallinity of materials through the diffraction phenomenon of X–ray in crystals. It is also an important means of exploring the structure of polymers and compounds. The crystalline state of polysaccharides determines the physical properties such as flexibility and solubility.

It can be clearly found from Supplementary Figure 2S that there are no wide diffraction peaks for APP60 and APP80 in the diffraction angle range of 10–55° and there exist obviously strong diffraction peaks, suggesting that there are some crystal fibers in APP60 and APP80, which is similar to the mixed fermentation polysaccharide studied by Lin et al. (2020). Compared with the ethanol concentration of 80%, the ethanol concentration of 60% precipitated 2θ and the decrease of peak intensity at 36 and 49° may be related to the testing environment, which indicates that the crystalline state of APP60 and APP80 basically remains the same, and the polysaccharide exists in a large number of crystalline forms. Besides, the increase of the ethanol volume fraction from 60 to 80% does not change the crystalline state of APPS, while in APP40 2θ, there is no obviously strong diffraction absorption peak in the range of 10°–55° while a wide diffraction peak exists. Furthermore, there is a large diffraction peak of steamed bread in the diffraction angle of 20°–30°, which indicates that APP40 polysaccharide is an amorphous polymer instead of a coexisting polymer of sub microcrystalline and amorphous. It is featured with crystal and amorphous structures. Besides, 2θ, the intensity of diffraction peaks at 36°, 49°, and so on decreased or even disappeared. The results demonstrated that the crystal structure of APPS was slightly damaged after 40% ethanol precipitation.

#### SEM and AFM Analysis

**Figure 7:**
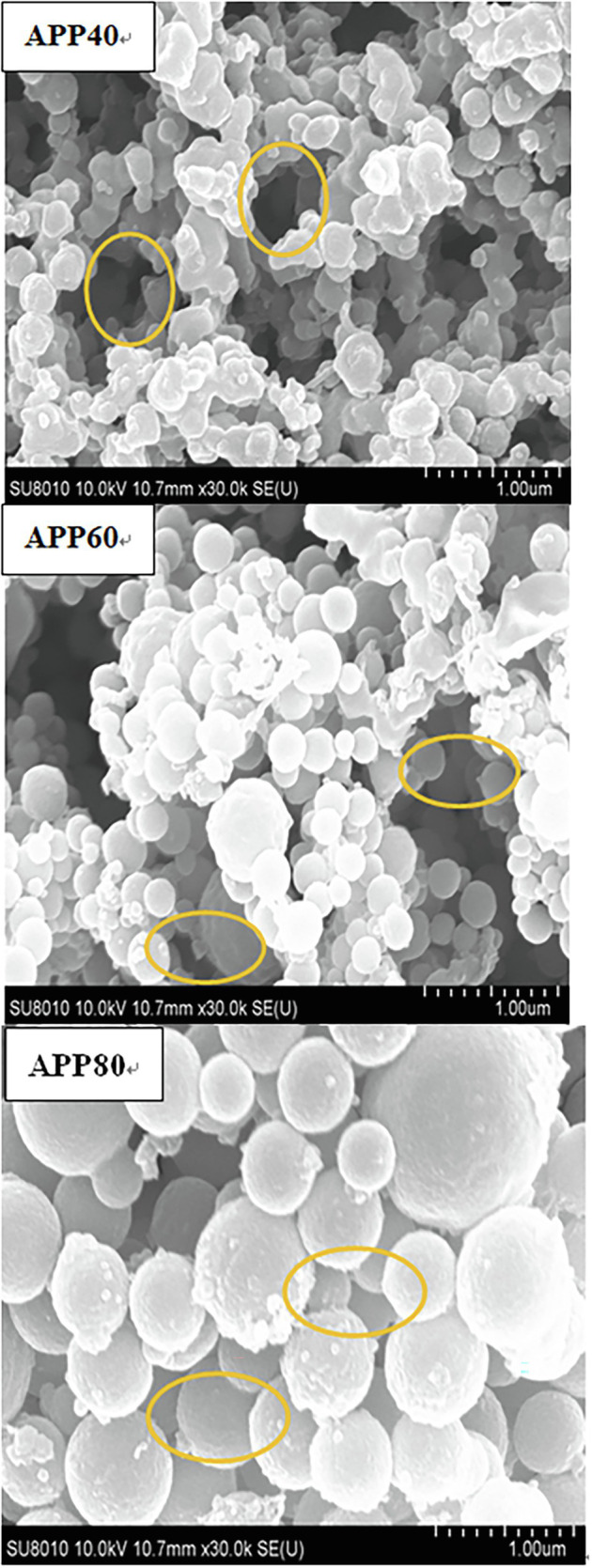
Scanning electron micrograph of precipitated at the ethanol concentration of 40% (APP40), precipitated at the ethanol concentration of 60% (APP60), and precipitated at the ethanol concentration of 80% (APP80).

Figure 7 presents APP 40, APP 60, and APP 80(×30k times). APP40 is flocculent or round, with irregular geometric shape, smooth, and uneven surface and wrinkled structure with holes, probably due to the utilization of protein and sugar in the fermentation process. The cellulase produced leads to the partial degradation of cellulose and hemicellulose, and the small particles on the surface are more dispersed (Tu et al., 2014). The results prove that there exists repulsive force between the molecules of polysaccharides and the attraction between them is small. Moreover, as repulsion force is more than gravity, there are holes, which may also be due to the aggregation of different types of beams by molecules or groups of molecules. As for the surface of APP60, it is mainly composed of small fragments, and there are many gaps between the fragments, probably because the increase of ethanol concentration reduces the intermolecular repulsion and increases the viscosity. The results show that the agglomerated large flake structure of APP80 is coarser, and the surface structure is more compact, whereas the particle size increases. The pores decrease or even disappear, and the molecules agglomerate.

**Figure 8:**
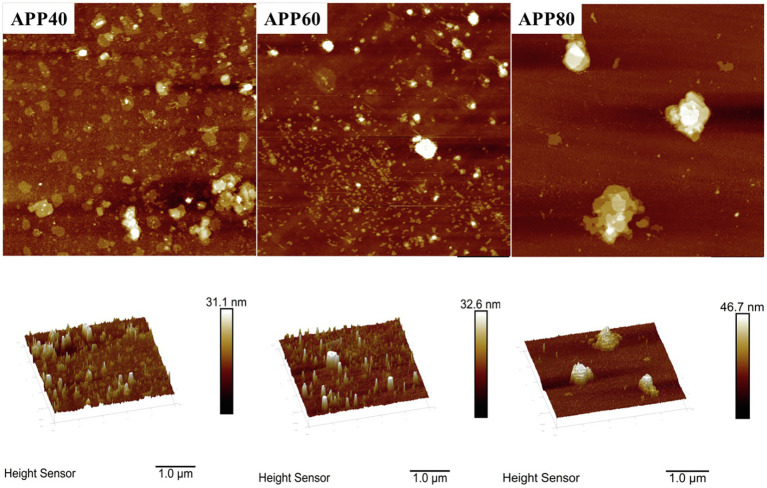
Atomic force micrograph of precipitated at the ethanol concentration of 40% (APP40), precipitated at the ethanol concentration of 60% (APP60), and precipitated at the ethanol concentration of 80% (APP80).

The gap structure of APPS was confirmed again by AFM (Figure 8). With the increase of ethanol concentration, the aggregation degree of polysaccharides increased, which was consistent with the conclusion from SEM. The height of APPS increased from 31.1 to 46.7 nm in AFM.

### Antioxidant Activity

#### DPPH Radical Scavenging Activity

2,2–diphenyl–1–picrylhydrazyl free radicals are stable ones that can accept electron or hydrogen free radicals to become stable antimagnetic molecules and have been extensively accepted as evaluators (Hu et al., 2004). The methanol solution of DPPH has a characteristic absorption maximum at 517 nm. Furthermore, the method of scavenging DPPH is to reduce the DPPH alcohol solution in the presence of hydrogen antioxidants by producing a non–free radical form of DPPH, since it can accommodate many samples in a short time and is sensitive enough to detect active ingredients at low concentrations.

**Figure 9:**
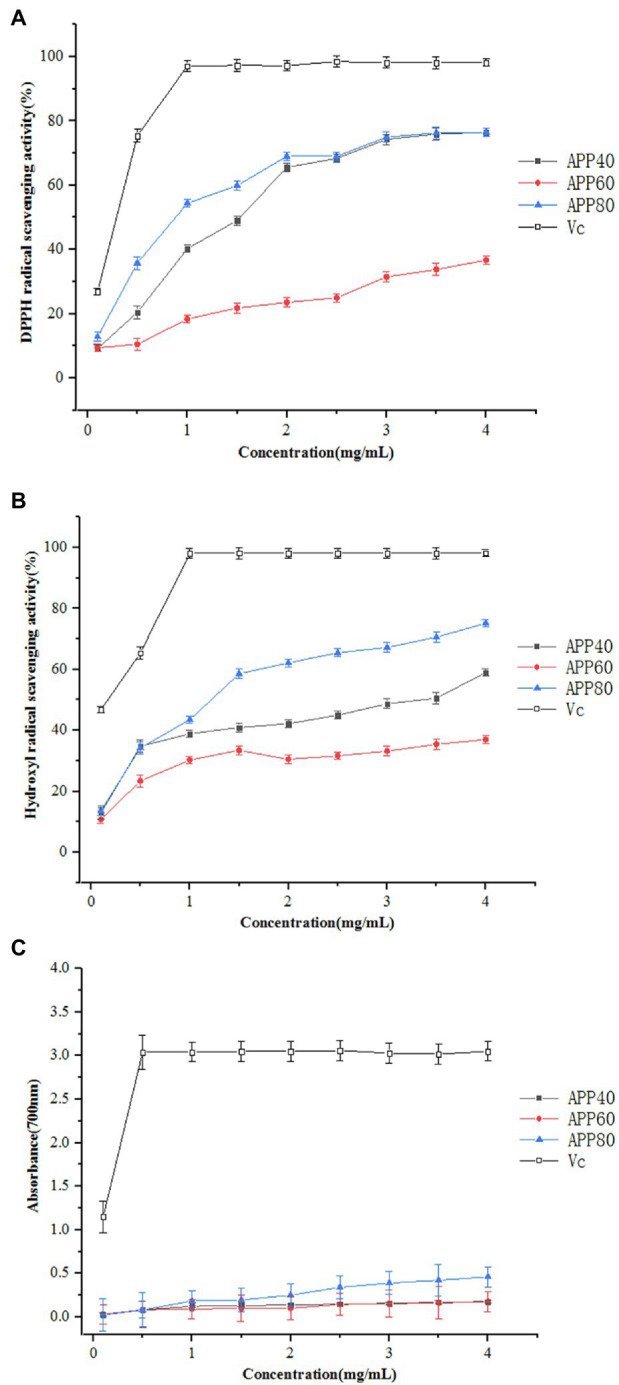
Antioxidant activity of polysaccharides. **(A)** DPPH radical scavenging activity; **(B)** hydroxyl radical scavenging activity; and **(C)** reducing power.

Following this principle, the scavenging effects of APP40, APP60, and APP80 on DPPH free radicals were determined. As shown in Figure 9A, the scavenging activity of polysaccharides on DPPH free radicals is related to the concentration of the sample. As the concentration increased, the scavenging activity of the test sample on DPPH was significantly enhanced. At a concentration of 1.5 mg/ml, the clearance rates of APP40, APP60, APP80, and *V*_c_ were 49.04, 21.80, 59.9, and 98.26%, respectively. The scavenging activity was similar to (Li et al., 2017). The DPPH free radical scavenging rate was mainly affected by the monosaccharide composition. In addition, it has been reported that the high molecular weight of polysaccharide may affect its solubility in water ethanol systems, thus affecting its scavenging ability (Xu et al., 2019). The above results indicate that the three polysaccharides may act as electron or hydrogen donors to scavenge DPPH.

#### Hydroxyl Radical Scavenging Activity

The results of APP40, APP60, and APP80 scavenging hydroxyl radicals are shown in Figure 9B. The scavenging effect of all samples on hydroxyl radicals is concentration-dependent, while the scavenging activity of hydroxyl radicals in descending order is APP80, APP40, and APP60. At 1 mg/ml, the scavenging activities of APP40, APP60, APP80, and *V*_c_ on hydroxyl radicals were 38.89, 30.26, 43.5, and 98.13%, respectively. APP40 and APP80 with their IC50 values of 3.48 and 1.29 mg/ml, respectively. The results were quite similar to the hydroxyl radical scavenging activity of polysaccharides from *Gynura procumbens* leaves reported by Li et al. (2017). In reactive oxygen, hydroxyl free radicals are considered an efficient oxidant that reacts with most biomolecules in living cells and causes serious damage to adjacent biomolecules. As a result, the removal of hydroxyl free radicals is important for the antioxidant defense of cells or food systems. Therefore, it is important to remove hydroxyl free radicals against oxidation.

#### Reducing Power

There exists a direct relationship between antioxidant activity and reducing capacity. Reductone is usually related to the presence of reduced ketones, which can provide a hydrogen atom, act as antioxidants by destroying free radical chains, and also react with certain precursors of peroxides to prevent the formation of peroxides. Antioxidation activity may be related to reduction ability and the compound can serve as an important indicator of its potential antioxidant activity. The reduction capacity of the three samples is shown in Figure 9C, revealing that the reduction ability of APP80 is the strongest, while that of APP40 and APP60 are slightly different. At a concentration of 1.5 mg/ml, the absorbance of APP40, APP60, and APP80 were .133, .097, and .194, respectively, while the reducing power data of APP40, APP60, and APP80 indicated that APP40, APP60, and APP80 may exert a role in the antioxidant effect.

## Conclusion

In brief, it was feasible to culture *A. polytricha* with yellow slurry water as the fermentation substrate. *Auricularia polytricha* polysaccharides with different relative molecular weight, molecular polarity, and pharmacological effects were separated and purified by alcohol precipitation, while the monosaccharide composition, molecular weight, and surface morphology of the three polysaccharides were different, which could provide a theoretical basis for the structural analysis and pharmacological effect exploration of APPS. Furthermore, it was found that the antioxidant capacity of each part of the polysaccharide was different, and that, among them, the APP80 low molecular weight polysaccharide showed the strongest antioxidant capacity. This study is of great significance to reduce environmental pollution and broaden the utilization of yellow slurry water. In the following experiments, by further improving the purity, the bonding mode and type of glyosidic bonds as well as isocarbon head structure were explored to clarify the structure–activity relationship of polysaccharides.

## Data Availability Statement

The original contributions presented in the study are included in the article/Supplementary Material, further inquiries can be directed to the corresponding author.

## Author Contributions

XZ and ZY served as experiment operator and wrote the main manuscript text. JW, JL, and FZ prepared Figures 1–9 and Table 2. HA, LH, and WZ modified articles. YH and YZ prepared Figures 1–9, Table 2 and modified articles. All authors contributed to the article and approved the submitted version.

## Funding

This research was financially supported by the Key Project in Agricultural of Guizhou Province in (2019) 2337 and 2379; (2022) key projects 013; and Guizhou Science and Technology Program [Qian Ke He Jichu (2019) 1071].

## Conflict of Interest

The authors declare that the research was conducted in the absence of any commercial or financial relationships that could be construed as a potential conflict of interest.

## Publisher’s Note

All claims expressed in this article are solely those of the authors and do not necessarily represent those of their affiliated organizations, or those of the publisher, the editors and the reviewers. Any product that may be evaluated in this article, or claim that may be made by its manufacturer, is not guaranteed or endorsed by the publisher.
