# Supplementary material for: High-Efficiency Production of Auricularia polytricha Polysaccharides Through Yellow Slurry Water Fermentation and Its Structure and Antioxidant Properties
Source: Front Microbiol. 2022 Feb 3;13:811275. doi: 10.3389/fmicb.2022.811275 (PMC8851468; doi:10.3389/fmicb.2022.811275)
Supplement: Supplementary file 1 [file Data_Sheet_1.docx]

Supplementary Fig.1S Fungus ball shape in the fermentation broth.

Supplementary Fig.2S X-ray diffraction diagrams of precipitated at the ethanol concentration of 40% (APP40), precipitated at the ethanol concentration of 60% (APP60), precipitated at the ethanol concentration of 80% (APP80).

1. Shape in fermentation medium


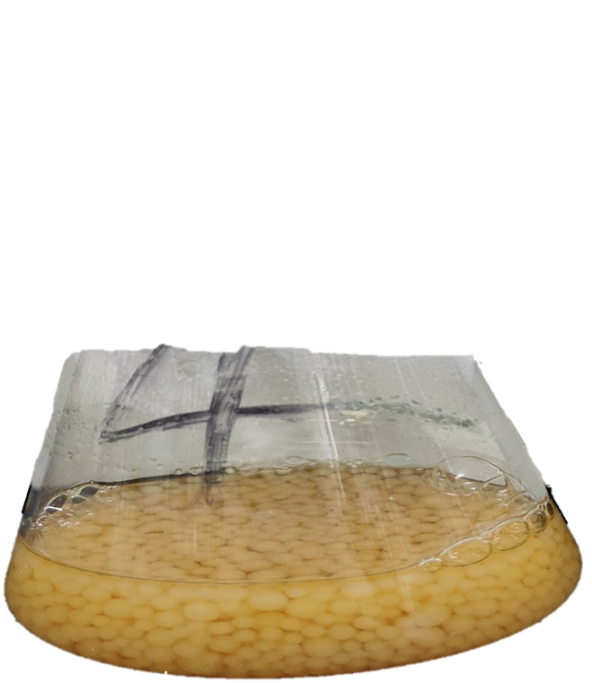


1. shape in yellow slurry water fermentation medium

Fig.1S


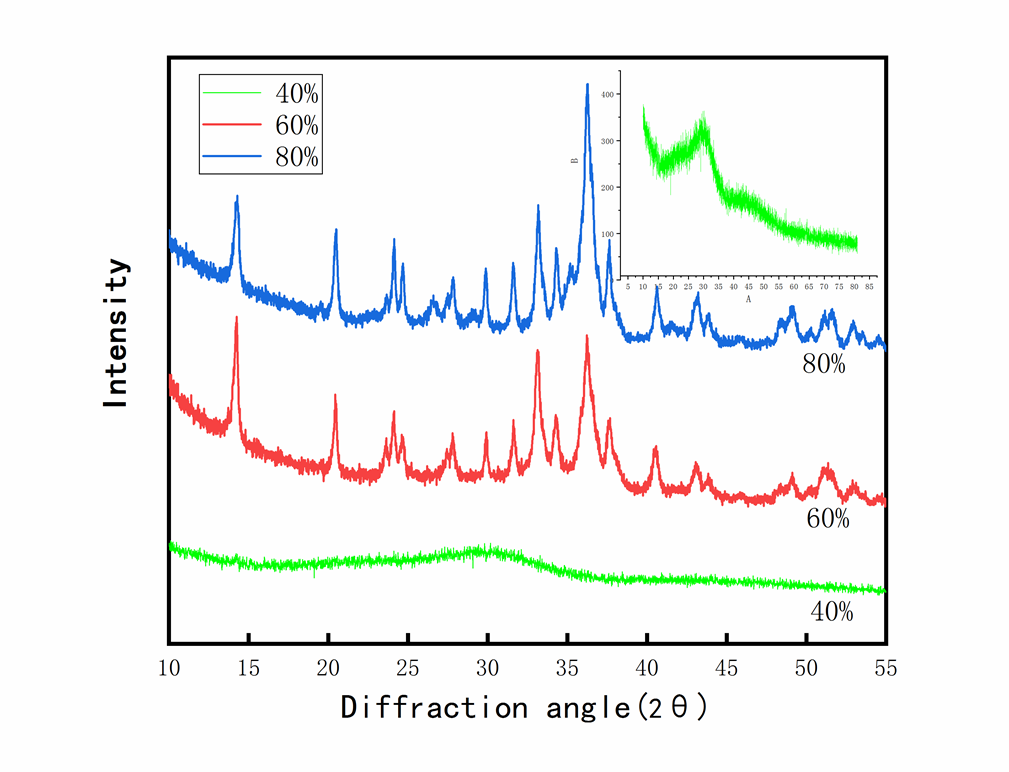


Fig.2S
